# Effect of mixed *Mycobacterium tuberculosis* infection on rapid molecular diagnostics among patients starting MDR-TB treatment in Uganda

**DOI:** 10.1186/s12879-023-08968-5

**Published:** 2024-01-10

**Authors:** Kevin Komakech, Lydia Nakiyingi, Ashab Fred, Beatrice Achan, Moses Joloba, Bruce J. Kirenga, Willy Ssengooba

**Affiliations:** 1 Department of Medical Microbiology, Mycobacteriology (BSL-3) Laboratory, Makerere University, Kampala, Uganda; 2 Department of Medicine, School of Medicine, Makerere University, Kampala, Uganda; 3 Department of Immunology and Molecular Biology, Makerere University, Kampala, Uganda; 4 Makerere University Lung Institute, Makerere University College of Health Sciences, Kampala, Uganda

**Keywords:** Mixed MTB infection, GeneXpert, LPA, Accuracy

## Abstract

**Background:**

Mixed *M. tuberculosis* (MTB) infection occurs when one is infected with more than one clonally distinct MTB strain. This form of infection can assist MTB strains to acquire additional mutations, facilitate the spread of drug-resistant strains, and boost the rate of treatment failure. Hence, the presence of mixed MTB infection could affect the performance of some rapid molecular diagnostic tests such as Line Probe Assay (LPA) and GeneXpert MTB/RIF (Xpert) assays.

**Methods:**

This was a cross-sectional study that used sputum specimens collected from participants screened for STREAM 2 clinical trial between October 2017 and October 2019. Samples from 62 MTB smear-positive patients and rifampicin-resistant patients from peripheral health facilities were processed for Xpert and LPA as screening tests for eligibility in the trial. From November 2020, processed stored sputum samples were retrieved and genotyped to determine the presence of mixed-MTB strain infection using a standard 24-locus Mycobacterial Interspersed Repetitive Unit–Variable Number Tandem-Repeat (MIRU-VNTR). Samples with at least 20/24 MIRU-VNTR loci amplified were considered for analysis. Agar proportional Drug Susceptibility Test (DST) was performed on culture isolates of samples that had discordant results between LPA and Xpert. The impact of the presence of mixed-MTB strain on Xpert and LPA test interpretation was analyzed.

**Results:**

A total of 53/62 (85%) samples had analyzable results from MIRU-VNTR. The overall prevalence of mixed-MTB infection was 5/53 (9.4%). The prevalence was highest among male’s 3/31 (9.7%) and among middle-aged adults, 4/30 (33.3%). Lineage 4 of MTB contributed 3/5 (60.0%) of the mixed-MTB infection prevalence. Having mixed MTB strain infection increased the odds of false susceptible Xpert test results (OR 7.556, 95% CI 0.88–64.44) but not for LPA. Being HIV-positive (*P = 0.04*) independently predicted the presence of mixed MTB infection.

**Conclusions:**

The presence of mixed-MTB strain infection may affect the performance of the GeneXpert test but not for LPA. For patients with high pre-test probability of rifampicin resistance, an alternative rapid method such as LPA should be considered.

**Supplementary Information:**

The online version contains supplementary material available at 10.1186/s12879-023-08968-5.

## Introduction

Tuberculosis (TB) remains a major cause of ill health and mortality globally [1]. The burden of TB is worsened by the development of drug-resistant *Mycobacterium tuberculosis* (MTB) strains. A total of 410 (370–450)/100,000 population were estimated to have developed multidrug-resistant or rifampicin resistant TB (MDR/RR-TB) in 2022 according to the World Health Organization (WHO) TB report [2]. In the same year, Uganda with an estimated population of 47 million people reported the incidence of TB of 198 (119–297)/100,000 population and the incidence of MDR/RR-TB of 3 (0.88–5.2)/100,000 population. Curbing the spread of drug resistant TB greatly relies on early case detection and the subsequent start of effective treatment. In most high TB burden settings, early case detection mostly depends on observed symptoms and chest radiography [3]. The confirmatory test, however, depends on sputum smear microscopy, mycobacterium culture and molecular GeneXpert MTB/RIF [4, 5]. In the case of rapid screening of multidrug-resistant (MDR) TB, Line Probe Assay (LPA) is always used as the confirmatory tests [6]. This makes the performance of rapid molecular diagnostic tests key in controlling MTB infection through rapid diagnosis and monitoring treatment response [7]. The main barriers faced by the rapid molecular tests for diagnosis of resistant TB is their inability to provide the full profile of resistance-conferring mutations and, for some like LPA, the need for specialized laboratory set-up [8]. In addition, mixed MTB strain infection, usually common in high TB burden settings, could also affect the performance of rapid molecular diagnostic tests [9].

Mixed MTB infection manifest when a TB patient is infected with more than one clonally distinct MTB strain [10]. The simultaneous transmission of multiple strains resulting in mixed infection may occur in populations of vulnerable individuals whereby both strains are able to bypass the host’s defense system and resist killing [11]. Super-infection can also occur when the severity of a current disease episode is such that it compromises the host innate immune response to a point that leads to increased susceptibility to infection with a secondary strain [11]. Alternatively, mixed-strain infections can arise if a subsequent infectious episode which was caused by a distinct strain result in relapse of the original infection yielding disease with two unique MTB strains that may have the same or different drug susceptibility profiles [12].

Hence, this form of mixed MTB infections has always caused false negative drug resistance profile of samples when phenotypic drug susceptibility tests (DST) are performed [13–15]. Mixed infections can also assist MTB strains to acquire additional mutations, facilitate the spread of drug-resistant strains and boost the rate of treatment failure [13, 16]. Several methods have been employed to detect mixed MTB infection though MIRU-VNTR has been the most widely used [16, 17]. It defines Mixed MTB infections by the presence of strains with different MIRU-VNTR patterns at two or more loci in the same sputum, lymph, or other samples from the same patient [16]. In some cases, fast-acquired drug resistance could also be caused by hetero-resistance which is the presence of both susceptible and resistant MTB strains in the same sample [18] . According to Cohen et al. (2012), this form of mixed MTB infections is particularly common in regions with a high rate of TB like Uganda. Therefore, this study aimed to determine the prevalence of mixed MTB infection and their implications to the interpretation of rapid molecular diagnostic test among patients being initiated on MDR-TB treatment at Mulago National TB treatment unit, Kampala, Uganda.

## Materials and method

### Study site and design

This was a cross-sectional study using pellets of sputum specimens collected from participants screened for STREAM 2 clinical trial between October 2017 and October 2019. All laboratory procedures were performed at Mycobacteriology (BSL-3) Laboratory at the Department of Medical Microbiology, Makerere University, Kampala, Uganda. From November 2020, processed stored sputum samples were retrieved and genotyped to determine the presence of mixed-MTB infection using a standard 24-locus Mycobacterial Interspersed Repetitive Unit–Variable Number Tandem-Repeat (MIRU-VNTR).

### DNA extraction

Briefly, 1 ml of the sputum pellet was re-suspended into 200 μl of Tris-EDTA buffer (10 mM Tris-HCl, 1 mM EDTA, pH 7.0) in a conical Eppendorf tube. The cell suspension was centrifuged at 13200 rpm for 5 minutes before re-suspending the pellet into 200 μl of Tris-EDTA (pH 7.0). The suspended pellet was incubated at 95 °C for a minimum of 1 hour using a Hybridization oven before re-centrifuging at 13000 rpm for 1 minute to pellet the cell debris. Finally, the supernatant containing the DNA was harvested and transfer into a sterile tube and stored at − 20 °C until further use [19].

### PCR amplification

Using the Genoscreen MIRU-VNTR Typing Kit, the 24 markers were amplified from purified DNA using 6 quadruplex PCR and fluorescent primers specific for the flanking regions of the targeted loci in the Pre-Amplification room. In the Amplification room, 2.0 μL of DNA extract and H37Rv DNA was added to each reaction tube and into the positive control tubes respectively. PCR tubes were then loaded into the PCR machine after ensuring they were tightly closed. PCR products were stored at 4 °C or - 20 °C until analysis [20].

### PCR product analysis and PCR product sizing

PCR product analysis was done using the electrophoresis capillary sequencer. Briefly, 2 μL of PCR product were migrated with a mixture of HiDi formamide and GeneScan 1200 LIZ on ABI 3730XL capillary sequencer to analyze and determine the PCR product size respectively. The experiment was setup as follows; Capillary length - 50 cm; Oven temperature - 63 °C; Injection voltage – 1.6 kV; Run voltage - 10 kV; Injection time - 25 s and Run time for 6500 s.

### Band interpretation of electrophorized products

From the capillary image peaks, the corresponding MIRU-VNTR peaks were interpreted as copy numbers based on the reference allele calling table in the Supply 2005 protocol [20]. Mixed-strain *M. tuberculosis* infections were categorized on the basis of the presence of multiple repeats at MIRU-VNTR loci which indicated genetic heterogeneity. Mixed infections were defined as the presence of ≥2 repeats at > 1 locus or presence of ≥2 repeats at 1 locus (not > 1 locus) in the same sputum sample. Single-strain infections were defined as those with single repeat patterns in all MIRU-VNTR loci.

### Strain identification using the MIRU-VNTR genotypes

To determine MTB strain lineages, relatedness or clustering, the MIRU-VNTR genotypes were matched with reference strains in the MIRU-VNTR*plus* database (http://www.miru-vntrplus.org/) using a categorical coefficient of 1 and a distance cut off of < 0.3 that corresponds to a seven-locus difference [21].

### Agar proportional method

This is the current gold standard method for drug susceptibility testing and was used to test the resistance/susceptibility status of isolates to two first-line drugs, Rifampicin (RIF) and Isoniazid (INH). This test was performed on Middlebrook 7H10 medium according to the Clinical and Laboratory Standards Institute (CLSI) procedures and recommended critical concentrations by World Health Organization (RIF-1.0 μg/mL, INH-0.2 μg/mL) [22, 23]. Bi-plates were used where drug concentrations were incorporated in the media to suit a final concentration of the respective drugs in each quadrant. An inoculum concentration of 1 McFarland was used, where 100 μl of it was dispensed on the plate and homogenously streaked before incubating the plates at 37 °C for 21 days. H37Rv laboratory strain was used as a positive control where it was inoculated on a drug free plate. After 21 days, any ≥1% growth on the drug containing plate compared to the drug free plate was interpreted as resistant and the reverse was true for a susceptible strain.

### Effect of tests

This was assessed by determining association between having mixed MTB infection and the rapid molecular diagnostic test results. Results were presented as proportions and percentages.

### Statistical analysis

Data obtained were coded and entered in the Microsoft Excel before analyzing using SPSS version 23. Descriptive analysis was used to summarize the data in form of frequencies and percentages. Mixed MTB infection was defined as presence of ≥2 repeats at > 1 locus or presence of ≥2 repeats at 1 locus (not > 1 locus) in the same sputum sample. Prevalence of mixed MTB infection was the frequency of patients with samples having mixed MTB strains over the total number of patient samples characterized. This was compared among patient’s characteristics and MTB lineages. A test result was categorized as “Affected” if it showed “Indeterminate” (the diagnostic test could not distinguish whether the sample was resistant or susceptible) or when it showed a “False Negative/Positive” result as compared to the reference comparator test – Agar Proportional Method (APM), in presence of mixed MTB infection. Bivariate analysis was conducted to measure an association between having mixed MTB infection and performance of rapid molecular diagnostic compared to the reference comparator. The risk factors of mixed MTB infection were also determined in a multivariate analysis for variable with *P*-Value < 0.02. Odds ratios (ORs) were used as the measure of association at 95% confidence intervals (CI) with *p*-values < 0.05 considered to be statistically significant.

## Results

A total of 62 patient MTB isolates were genotyped, of which 53 (85.5%) were characterized. The overall prevalence of mixed MTB infection was 5/53 (9.4%). Mixed MTB infection prevalence was 3/5 (60.0%) among males and was highest among middle-aged adults 4/5 (80.0%). Among patients with mixed MTB infection, 3/5 (60.0%) were from MTB Lineage 4. Mixed MTB infection did not significantly vary by patient characteristics or MTB lineage (*P* > 0.05), (Table 1).

**Table 1:** Descriptive characteristics of participants and prevalence of mixed MTB infection

Characteristics		Frequency(*N* = 53)	Percentage	Frequency of Mixed TB infection (*N* = 5)	Percentage of Mixed TB infection	*P*-Value
**Sex**	Male	31	58.5	3	60.0	0.831
	Female	22	41.5	2	40.0	
**Age**	Young adults (18–30)	18	34.0	1	20.0	0.240
	Middle age adults (31–45)	30	56.6	4	80.0	
	Old age adults (> 45)	5	9.4	0	0.0	
**MTB Lineage**	1	2	3.8	0	0.0	0.388
	2	4	7.5	1	20.0	
	3	14	26.4	1	20.0	
	4	33	62.3	3	60.0	

### Effect of mixed MTB infection on the performance of rapid molecular diagnostic tests

Line Probe Assay detected 47% (25/53) and 36% (19/53) of the isolates as MDR-TB and RR strains respectively. In addition, it also reported 8% (4/53), 2% (1/53), and 6% (3/53) of the isolates as isoniazid-resistant, indeterminate, and sensitive to both isoniazid & rifampicin respectively. GeneXpert reported 90% (48/53), 6% (3/53), and 4% (2/53) of the isolates as RR, indeterminate, and sensitive to both isoniazid & rifampicin respectively. Using Agar Proportional Method (APM), 57% (30/53) and 43% (23/53) of the isolates were MDR-TB and rifampicin-resistant respectively (Table 2).

**Table 2:**
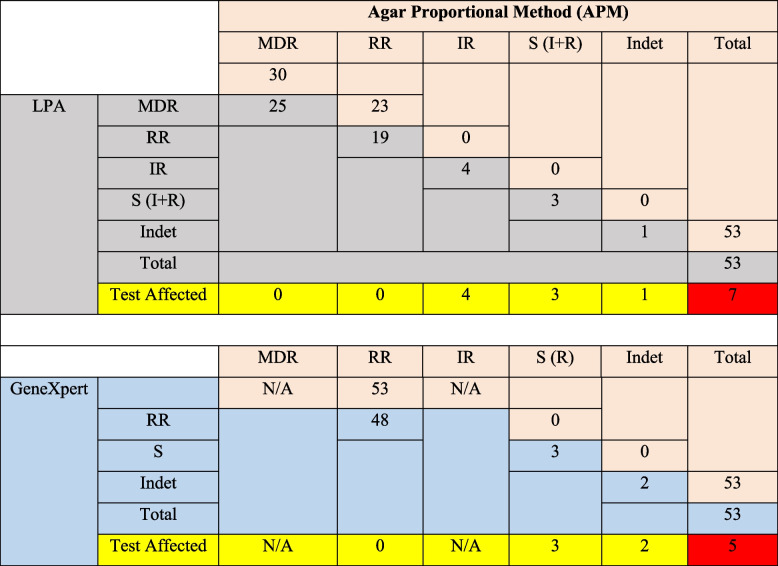
Comparison of APM results with LPA and GeneXpert test results

*MDR* Multidrug Resistant: *R* Rifampicin Resistant: *IR* Isoniazid Resistant: *I* Isoniazid: *R* Rifampicin: *S* Sensitive: *Indet* Indeterminate: *LPA* Line Probe Assay: *N/A* Not Applicable (Pink cells represent results from APM-Reference standard, Grey cells represent results from LPA, Blue cells represent results from GeneXpert MTB/RIF, Yellow cells represent number of tests affected based on results from the reference standard and Red cells represent total number of tests affected by each rapid molecular method)

Having mixed MTB infection increased the odds of false susceptible Xpert test results (OR 7.556, 95% CI 0.88–64.44) but not for LPA (Table 3); and being HIV-positive (OR 24.00, *P = 0.04 95% CI* 1.656–347.89) independently predicted presence of mixed MTB infection (Table 4).

**Table 3:** Bivariate analysis of LPA and XPT results affected due to mixed MTB infection

	Variable	% participants *N* = 53		Frequency (n)	LPA/XPT results Affected n (%)	unadjusted OR (95% CI)	*P*-Values
LPA	Mixed TB infection	No	90.6	48	5/48 (10.4)	1.071 (0.103–11.130)	0.954
		Yes	9.4	5	2/5 (40.0)		
XPT	Mixed TB infection	No	90.6	48	2/48 (4.2)	7.556 (0.886–64.445)	0.039*
		Yes	9.4	5	3/5 (60.0)		

*LPA* Line Probe Assay: *XPT* Xpert MTB/RIF: *OR* Odds Ratio: *CI* Confidence Interval, *adjusted OR was not possible, only one variable met the criteria

**Table 4:** Factors associated with mixed MTB infection among MDR/RR-TB patients

Variable		Participants (n/%) *N* = 53	Frequency (n)	Positive for Mixed TB infection n (%) *N* = 5	Unadjusted* OR (95% CI)	*P*-Values
**MDR**	No	54.8	23	2/23 (8.7)	1.071 (0.103–11.130)	0.954
	Yes	45.2	30	3/30 (10.0)		
**XPT test results**	RR D	92.9	48	3/48 (6.3)	24.00 (1.66–347.89)	0.033*
	RR ND	7.1	5	2/5 (40.0)		
**HIV**	Negative	61.9	37	1/37 (2.7)	8.333 (0.84–82.86)	0.040*
	Positive	38.1	16	4/16 (25.0)		

*RR* Rifampicin Resistance: *ND* Not Detected: *D* Detected: *XPT* Xpert MTB/RIF: *MDR* Multi-Drug Resistant: *OR* Odds Ratio: *CI* Confidence Interval: *few variables for adjusted OR

## Discussion

Our study done among MDR/RR-TB patients initiating treatment, the prevalence of mixed MTB infection was 9.4%. Having mixed MTB infection affected the performance of Xpert test assays but not for LPA. Mixed MTB infection was associated with HIV and false rifampicin susceptible results by Xpert. The high prevalence of mixed MTB infection was comparable to 7.1% which was reported by Dickman et al. (2010) [24] and higher than the 4% reported by Ssengooba et al. (2015) [17]. The difference in the prevalence could be attributed to the detection method used or the type of sample used. This is because Dickman et al. (2010) amplified 15 MIRU-VNTR loci using a single target ordinary PCR which is inferior for the detection of mixed MTB infection compared to amplifying 24 MIRU-VNTR loci which has a higher resolution [25]. Ssengooba et al. (2015) used blood and sputum samples from patients who were none MDR/RR-TB but with advanced HIV/AIDS. Hence, the prevalence could be lower since Hanekom et al. (2013) [26] reported a higher prevalence of 15% among MDR-TB patients in South Africa. However, this prevalence is slightly lower compared with that of 11.1% reported by Muwonge et al. (2014) [27]. The prevalence was highest among middle-aged adults and within Lineage 4 though none of the above variables were significantly associated with it. These findings agree with Sobkowiak et al. (2018) [28] who reported no association of mixed MTB infection with age and sex. However it disagrees with Pang et al. (2015) [29] who reported an association between mixed MTB infection and age and sex. In addition, Pandey et al. (2020) [30] also reported strong association between having mixed MTB infection and CAS1_Delhi lineage (Lineage 3) which disagrees with this findings. The difference in these findings could be attributed to the geographical location as Lineage 4 is the dominant lineage in Uganda. Studies have found that different MTB lineages are geographically located except Lineage 4 which seems to be widely distributed [31].

GeneXpert MTB/RIF and LPA were the rapid diagnostic tests whose performances were assessed in the presence of mixed MTB infection. The study showed that having mixed MTB strain infection increased the odds of affecting GeneXpert results by either giving indeterminate results or false results. This finding is in agreement with [25, 32, 33] who reported that the presence of a mixture of resistant subpopulations may create difficulties in the interpretation of rapid molecular drug resistance tests because it leads to ‘indeterminate’ test results. However, there was no significant association between having mixed MTB infection and having LPA test results affected. This could be because LPA detects resistant MTB subpopulations when they comprise ≥5% of the bacilli in the sample [34] unlike with GeneXpert which requires > 50% of the resistant subpopulation for its detection [16, 35–37]. Hence the presence of minority-resistant strains was most likely missed out by the GeneXpert test.

Risk factors associated with mixed MTB infection were also assessed. Overall, participants that had MDR-TB 10.0% (3/30), middle age adults (31–45) 13.3% (4/30), and those that were HIV positive 25.0% (4/16) had more cases of mixed MTB infection (Table 4). This findings agree with Pang et al. (2015) [29] who reported that mixed MTB infections were significantly more likely to occur in middle age adults than in other age groups. However, this finding disagrees with [28, 38] who reported no significant association between having HIV and mixed MTB infection. However, it agrees with Dickman et al. (2010) [24] and with Ssengooba et al. (2015) [17] who reported significant association between having HIV and mixed MTB infection. This finding is also in agreement with Naidoo et al. (2018) [39] who reported recurrent TB infections among HIV positive patients being associated with mixed MTB infection. The study also showed that having MDR-TB did not predict the presence of mixed MTB infection which is in agreement with Shin et al. (2018) [40] who reported no association between mixed MTB infection and having MDR-TB. Testing sensitive for rifampicin resistance by GeneXpert given you have MDR/RR TB by culture increased the odds of having mixed MTB infection. The inefficiency of GeneXpert in detecting rifampicin resistance has been reported by several studies [41]. The major cause of false results could be due to the presence of rifampicin-resistant strains at low populations which is undetectable by GeneXpert [41]. It could also be due to the presence of rifampicin mutations that occur outside the rifampicin resistance detectable regions [42]. In addition, this could also be due to the presence of disputed mutations which can only be expressed phenotypically but not Genotypically according to Torrea et al. [43] and Eddabra & Neffa [44].

The main implication of this finding is that mixed MTB infection affects Xpert tests, despite it being used as the main rapid molecular point of care test for TB in Uganda. Further studies should evaluate what form of mixed MTB infection affects Xpert tests. Since we used stored samples, this could have impacted on the accuracy of the tests used, however, since these are DNA-based, this could be minimal to significantly affect our findings. The strength of our study is that we used sputum samples for determining mixed MTB infection. A previous study found that culture alters the mycobacterial population and underestimates the prevalence of mixed infection [45].

## Conclusion

The presence of mixed-MTB infection may affect the performance of the GeneXpert test but not for LPA. In determining resistance among MTB patients, rapid resistance-determining methods may complement each other. In case of inconclusive results among patients with high pre-test probability, an alternative rapid method, possible LPA should be considered.

### Supplementary Information

**Additional file 1.**

## Data Availability

The datasets generated during the current study are available under supplementary material.
